# Diagnosis of pathogens causing bacterial meningitis using Nanopore sequencing in a resource-limited setting

**DOI:** 10.1186/s12941-022-00530-6

**Published:** 2022-09-05

**Authors:** Srinivas Reddy Pallerla, Do Van Dong, Le Thi Kieu Linh, Trinh Van Son, Dao Thanh Quyen, Phan Quoc Hoan, Ngo Tat Trung, Nguyen Trong The, Jule Rüter, Sébastien Boutin, Dennis Nurjadi, Bui Tien Sy, Peter G. Kremsner, Christian G. Meyer, Le Huu Song, Thirumalaisamy P. Velavan

**Affiliations:** 1 Institute of Tropical Medicine, Universitätsklinikum Tübingen, Wilhelmstrasse 27, 72074 Tübingen, Germany; 2 Vietnamese-German Center for Medical Research, VG-CARE, Hanoi, Vietnam; 3 Department of Molecular Biology, 108 Military Central Hospital, Hanoi, Vietnam; 4 Department of Infectious Diseases, Medical Microbiology and Hygiene, Heidelberg University Hospital, Heidelberg, Germany; 5 Department of Infectious Diseases and Microbiology, University of Lübeck, Lübeck, Germany; 6 Department of Microbiology, 108 Military Central Hospital, Hanoi, Vietnam; 7 Centre de Recherche Médicales de Lambaréné, Lambaréné, Gabon; 8 108 Military Central Hospital, Hanoi, Vietnam

**Keywords:** Nanopore, Bacterial meningitis, Vietnam, 16S rRNA, Next-generation sequencing, Diagnosis and pathogen genome

## Abstract

**Aim:**

The aim of the present study is to compare the performance of 16S rRNA Nanopore sequencing and conventional culture in detecting infectious pathogens in patients with suspected meningitis in a resource-limited setting without extensive bioinformatics expertise.

**Methods:**

DNA was isolated from the cerebrospinal fluid (CSF) of 30 patients with suspected bacterial meningitis. The isolated DNA was subjected to 16S sequencing using MinION™. The data were analysed in real time via the EPI2ME cloud platform. The Nanopore sequencing was done in parallel to routine microbiological diagnostics.

**Results:**

Nanopore sequencing detected bacterial pathogens to species level in 13 of 30 (43%) samples. CSF culture showed 40% (12/30) positivity. In 21 of 30 patients (70%) with suspected bacterial meningitis, both methods yielded concordant results. About nine of 30 samples showed discordant results, of these five were false positive and four were false negative. In five of the culture negative results, nanopore sequencing was able to detect pathogen genome, due to the higher sensitivity of the molecular diagnostics. In two other samples, the CSF culture revealed Cryptococcus neoformans and Streptococcus pneumoniae, which were not detected by Nanopore sequencing. Overall, using both the cultures and 16S Nanopore sequencing, positivity rate increased from 40% (12/30) to 57% (17/30).

**Conclusion:**

Next-generation sequencing could detect pathogens within six hours and could become an important tool for both pathogen screening and surveillance in low- and middle-income countries (LMICs) that do not have direct access to extensive bioinformatics expertise.

**Supplementary Information:**

The online version contains supplementary material available at 10.1186/s12941-022-00530-6.

## Introduction

Bacteria, viruses, fungi and protozoa are common causes of sepsis and meningitis. The majority of cases result from bacterial infections. It is estimated that 48.9 million cases and 11 million of the sepsis-related fatalities reported worldwide in 2017 accounted for 20% of all global deaths [1]. Further, meningitis caused an estimated 250,000 deaths in 2019 [2]. The infections are highly prevalent in low and middle-income countries (LMIC), and the lack of infrastructure and appropriate diagnostic facilities leads to the high morbidity and mortality [3, 4]. Both bacterial meningitis and septic shock are life-threatening emergencies; therefore, quick and accurate diagnoses are essential. Cerebrospinal fluid (CSF) and blood cultures are the diagnostic gold standards; however, cultures are time-consuming. Any delay in the diagnosis increases mortality and hospital stays, but also health care expenditures [5–7]. Several studies that have compared the yield of bacterial cultures with PCR-based diagnosis have found that 30–50% of culture-negative CSF specimens are positive when applying molecular tools [8, 9]. These discrepancies may be explained by the empiric use of antibiotics, a low bacterial count, low blood volume, or non-culturable and fastidious organisms that require special growth conditions [10]. RT-PCR for detecting bacteria are now rapidly being used and offer a quick alternative in the timely diagnosis of a causative pathogen, thus reasonably complementing culture-based microbiological diagnostics.

Recently, several commercial 16S PCR platforms have been made available for sepsis diagnosis, including Universal digital high-resolution melt (U-dHRM), SepsiTest™, LightCycler™ SeptiFast, and the IRIDICA system [11]. The initial hardware and recurring expenses are high, rendering these systems largely unaffordable in most LMIC. The 16S rRNA gene has proven to be useful in diagnosing bacterial infections [6, 10]. Next-generation sequencing (NGS) technologies are now becoming an important diagnostic tool in detecting infectious diseases, directly from patient samples [12–14]. The Oxford Nanopore Technologies (ONT) NGS is gaining attention as it has several advantages compared to other sequencing platforms. It is a portable device, requires manageable financial investment and low maintenance costs. Moreover, it provides a rapid turnaround time with user-friendly EPI2ME cloud-based bioinformatics pipelines [12]. A previous disadvantage of this technology was that it had a high sequencing error rate of 5–15% [15]. Meanwhile the technology has improved significantly with far lower error rate, i.e.,  < 3.5% [16]. High throughput sequencing using the Flongle™ flowcell will considerably reduce the costs per sample and thus allowing the use of such technology in a LMIC setting.

Rapid identification of infectious disease and access to their genomes is essential for establishing successful therapeutic and preventive measures [17]. Several studies have shown that the portable Nanopore sequencing system can be used for rapid and accurate diagnosis of bacterial meningitis, sepsis, and other infections from a variety of samples in both developed and LMIC countries [18–24].

The Nanopore system has been used extensively for infectious disease surveillance and diagnosis. It has been used in low-resource settings to monitor and sequence ZIKA, EBOV, and SARS-CoV-2 viral genomes during outbreaks in Brazil, Guinea, and worldwide [25–28]. Despite these successes of nanopore sequencing, limitations remain, such as the limited sample size used in most studies and the use of in-house bioinformatics pipelines that require bioinformatics expertise [18, 19]. Some of these studies were performed with positive blood cultures rather than directly with clinical samples [20, 21], which is time consuming. Since only a few studies have been conducted to date, further studies are needed to validate the Nanopore system and assess its sensitivity, specificity, and utility in clinical applications.

The aim of the present study was to use the Nanopore MinION™ NGS tool for direct diagnosis and genotyping of bacterial meningitis in patients with suspected bacterial meningitis in CSF samples based on 16S rRNA sequencing using the Nanopore EPI2ME cloud without specific on-site bioinformatics expertise. The 16S rRNA results shall be compared with culture methods routinely used to screen for pathogens.

## Methods and materials

### Study cohort

The retrospective hospital-based cohort study was collected between 2019 and 2020 at 108 Military Central Hospital, a referral hospital for adults with a catchment area including Hanoi and the northern provinces of Vietnam. Microbiological examination was performed by microscopy of CSF according to the hospital routine standards. All recruited patients were admitted to the infectious diseases department and aged 15 years or older, with symptomatic meningitis based on criteria for a central nerval system (CNS) infection and eligible for inclusion [29]. The inclusion criteria were clinical signs of CNS infection such as fever, headache, vomiting, neck stiffness, elevated C-reactive protein, procalcitonin, white blood cell count, focal neurological signs including seizures and altered consciousness, and/or elevated white blood cell count (WBC) in CSF. Patients with other etiologies as a cause for the neurological disorder were excluded.

The diagnostic results of the CSF from the patients in the study were available from the routine microbiological diagnostics. However, the diagnostic results were blinded until Nanopore sequencing was completed. In this retrospective study we included specimens from 30 patients with the clinical diagnosis of bacterial meningitis based on routine laboratory parameters summarized in Table 1. Two of the 30 (7%) patients with suspected bacterial meningitis had lethal outcome.

**Table 1:** Baseline characteristics of patients with suspected bacterial meningitis

Characteristics^a^	CSF (n = 30)
Age (years)	49 (19–82)
Male	29
Female	1
CSF neutrophils (%)	93 (77–97)
CSF leucocytes (/mm^3^)	1080 (474–4335)
CSF glucose (mmol/L)	1.1 (0.2–2.3)
CSF protein (g/L)	3.2 (1.9–4.7)
Blood—WBC (g/L)	17 (12–21)
Blood—neutrophils (%)	87 (81–92)
Procalcitonin (ng/ml)	7.1 (0.8–13.7)
Recovered/deceased	28/2

Values shown are median with interquartile ranges in brackets

*CSF* cerebrospinal fluid; *WBC* white blood cells

^a^10 missing values for CSF neutrophils, 11 missing values for CSF leukocytes, 9 missing values for CSF glucose, CSF protein, blood WBC and blood neutrophils, 12 missing values for procalcitonin

For microbiology cultures and molecular assessment, at least 2 mL of CSF was obtained by lumbar puncture. Samples were stored at − 80 °C until further use. According to international standards, clinical cases were categorized in three distinct groups by CSF values [29]. Bacterial CNS infection were defined by WBC > 10 predominance of polymorphonucleocytes, protein > 1 g/L, and glucose < 40 mg/dL; aseptic/viral CNS infection (VI) defined by WBC > 10, predominance of lymphocytes, protein < 2 g/L, and normal glucose levels; tuberculous CNS infection defined by WBC > 10, predominance of lymphocytes, protein > 1 g/L, and glucose < 40 mg/dL.

Diagnostic procedures and treatment of patients followed the clinical routine. Demographic and clinical data, routine hematology, and clinical biochemistry parameters as well as blood culture results were recorded. In addition, computed tomography (CT) or magnetic resonance imaging (MRI) was performed. The CSF patients were treated with ceftriaxone, meropenem, vancomycin, colistin, levofloxacin, and moxifloxacin antibiotics. Sepsis patients were treated with levofloxacin, amikacin, moxifloxacin, linezolid, vancomycin, ceftazidime, piperacillin-tazobactam, tiepenem, meropenem, cefoxitin, doripenem, and ampicillin-sulbactam. In total 30 samples from 30 patients were included in the study.

### Microbiological diagnostic of CSF culture

The microbiological diagnostic was performed in the routine diagnostics following the current diagnostical standards. Identification of bacterial species was performed using the VITEK^®^ 2 automated system (BioMérieux, Craponne, France).

### DNA isolation

DNA was extracted from one mL of CSF from patient samples using the SaMag bacterial DNA extraction kit (Sacace Biotechnologies S.r.l., Como, Italy) [30]. Human DNA in the samples was depleted using mammalian cell lysis buffer 1 (2 M Na_2_CO_2_ pH 9.8, 1% Triton-X100) before proceeding with the SaMag extraction kit as described previously [31]. DNA quality and quantity was assessed with the Qubit™ 4 fluorometer using the Qubit™ dsDNA BR Assay Kit (Thermo Fisher Scientific, Waltham, MA, USA).

### 16S rRNA sequencing with nanopore MinION™

For multiplex sequencing, the16S rRNA gene was PCR-amplified from CSF sample DNA using the 16S barcoding kit SQK-16S024 (Oxford Nanopore Technologies, Oxford, UK) following the manufacturer’s instructions. The 16S barcoding kit includes 16S primers covering full-length 16SrRNA (~ 1500 bp) (16S-27F—AGRGTTTGATYHTGGCTCAG 16S-1492R-TACCTTGTTAYGACTT) [32, 33]. The PCR was performed using 10 μL of DNA sample (concentration range < 1–50 ng/µL) per reaction, including a blood culture positive control of an *Acinetobacter baumannii* strain and a non-template control. Amplicons were purified using AMPure XP beads, quantified using the Qubit™ 4 fluorometer with the Qubit™ dsDNA BR Assay Kit (Thermo Fisher Scientific, Waltham, MA, USA), and pooled having total 100 ng of the 16S PCR amplicons in 10 µL (5 ng /sample). Sequencing was performed with the R9.4.1 MinION™ system for six hours (Oxford Nanopore Technologies, Oxford, UK) and data collected using fast-basecalling by the MinKNOW software (v.18.01.6). Data generated by MinKNOW was analyzed in real-time on the Nanopore EPI2ME cloud. The EPI2ME analysis was performed using complete sequencing data generated from sequencing run and 200 NGS reads/sample, and the read length cut-off was set to min 1400 bp to max 1700 bp (sequencing statistics are summarized in Additional file 1: Table S1).

## Results

### Pathogens identified by CSF culture and blood culture

Of the 30 CSF specimens, 12 (40%) yielded positive result from CSF cultures and the pathogens included *Streptococcus pneumoniae* (n = 4), *Streptococcus suis* (n = 4), *Streptococcus mitis* (n = 1), *Pseudomonas aeruginosa* (n = 1), *Aeromonas Sobria* (n = 1) and *Cryptococcus neoformans* (n = 1). *S. pneumoniae* and *S. suis* were the most common pathogen (33%; 4/12 for each species) identified by CSF culture. In 5/30 (17%) patients, the same bacterial species were also detected in blood culture, they included: *S. pneumoniae* (n = 2), S. suis (n = 3), and *Staphylococcus xylosus* (n = 1).

### Pathogens identified using 16S Nanopore sequencing

On screening the 30 patient specimens for pathogenic bacteria, we observed a 16S-PCR positivity of 43% (13/30) using the Oxford Nanopore 16S Barcoding Kit (SQK-16S024). EPI2ME cloud data analysis results were identical when using all reads generated per sample (Additional file 1: Table S1) or using only the first 200 reads per sample. Therefore, our data suggests that 200 reads/sample may be sufficient for reliable pathogen detection. Further reduction to 100 reads per sample would also have produced identical results. However, we arbitrarily retained the 200 reads per sample. The pathogens identified include *S. suis* (n = 5), *S. pneumoniae* (n = 4), *Enterococcus hirae* (n = 1), *Neisseria gonorrhoeae* (n = 1), *Klebsiella pneumoniae* (n = 1), *Aeromonas jandaei* (n = 1). Multiplex sequencing of 16S PCR amplicons from all samples in a single run with Nanopore MinION sequencing and real-time NGS data analysis on EPI2ME Cloud detected bacterial pathogens at the species level within one hour. The DNA concentration for each sample is summarized in Table 3. The bacterial species identified by Nanopore sequencing of all the 16S PCR positive samples are summarized in Table 2. *S. suis* and *S. pneumoniae* were the most common pathogen (5/30; 17% and 4/30; 13%, respectively) detected using this method.

**Table 2:** Nanopore MinION sequencing results

Sample ID	Pathogen	MinION reads^b^ Total (Predominant %)
C3	*Streptococcus pneumoniae*	184 (94)
C4	*Streptococcus suis*	187 (87)
C7	*Streptococcus pneumoniae*	187 (94)
C8	*Streptococcus pneumoniae*	182 (96)
C10	*Streptococcus suis*	178 (94)
C11	*Streptococcus suis*	166 (97)
C13	*Enterococcus hirae*	182 (57)
C17	*Streptococcus pneumoniae*	164 (91)
C23	*Neisseria gonorrhoeae*	219 (98)
C25^a^	*Streptococcus suis*	175 (94)
C26	*Streptococcus suis*	190 (94)
C27	*Klebsiella pneumoniae*	173 (83)
C30	*Aeromonas jandaei*	178 (75)
Positive control	*Acinetobacter baumannii*	156 (94)

^a^Sample was run in a separate sequencing run (not part of the single pool)

^b^200 reads/sample were considered for analysis

### Comparison of conventional microbiology and Nanopore sequencing

Overall, 21 of 30 CSF samples displayed concordant results between Nanopore sequencing and conventional culture. Nanopore sequencing was able to detect eight of 12 (67%) culture positive samples correctly. Nine of thirty (30%) samples showed discordant results, of these five were culture negative and yielded positive signal in the Nanopore and four were culture positive but yielded negative signal in the Nanopore. In five of the 18 (28%) culture negative results, nanopore yielded positive signals, indicating that the Nanopore sequencing was able to detect pathogens in additional samples, due to the higher sensitivity of the 16S PCR. Overall, using both the cultures and 16S Nanopore sequencing, positivity rate increased from 40% (12/30) to 57% (17/30). In four of 12 samples, Nanopore did not yield positive results for *S. pneumoniae* (C7 and C14), *P. aeruginosa* (C12) and *C. neoformans* (C19). In the two culture-positive and Nanopore-negative samples, *S. pneumonia* were plausible since *S. pneumoniae* were also detected in the blood culture of these patients. For the culture-positive Nanopore-negative with *P. aeruginosa* (C12), the CSF culture and the blood culture delivered diverging results so that the reliability and the plausibility of this results is questionable. In sample C19, a Nanopore-negative and culture-positive results was expected since *C. neoformans* is a fungus and thus would not be detected by 16S-based sequencing. For samples C6 and C30, the culture and Nanopore sequencing results were divergent at the species level but not the genus level. A potential explanation may be the limited accuracy of biochemical identification to the species level. However, since the genus of these bacteria were concordant and thus these were interpreted as concordant results (Table 3).

**Table 3:** Comparison of detected bacterial species by Nanopore and conventional culture

ID	Sex	Age	Diagnosis	DNA (ng/µL)	Nanopore	CSF culture	Blood culture (data from routine microbiology)
C1	M	53	Bacterial meningitis	< 1	N	N	N
C2	M	19	Bacterial meningitis, Nephrotic syndrome	< 1	N	N	N
C3	M	54	Bacterial meningitis	51	***S. pneumoniae***	***S. pneumoniae***	N
C4	M	41	Bacterial meningitis	29	*S. suis*	N	N
C5	M	37	Bacterial meningitis	18	N	N	N
C6	M	21	Bacterial meningitis	47	***S. pneumoniae***	***S. mitis***	N
C7	M	37	Bacterial meningitis	28	N	*S. pneumoniae*	*S. pneumoniae*
C8	M	30	Bacterial meningitis/brain trauma	12	*S. pneumoniae*	N	N
C9	M	44	Bacterial meningitis	10	N	N	N
C10	M	70	Bacterial meningitis	5	***S. suis***	***S. suis***	***S. suis***
C11	M	59	Bacterial meningitis	17	***S. suis***	***S. suis***	N
C12	M	73	Brain abscess after surgery	24	N	*P. aeruginosa*	*S. xylosus*
C13	M	51	Bacterial meningitis	39	*E. hirae*	N	N
C14	M	30	Bacterial meningitis	17	N	*S. pneumoniae*	*S. pneumoniae*
C15	M	82	Bacterial meningitis	8	N	N	N
C16	M	60	Bacterial meningitis	12	N	N	N
C17	M	20	Bacterial meningitis	33	***S. pneumoniae***	***S. pneumoniae***	N
C18	M	30	Bacterial meningitis	45	N	N	N
C19^a,b^	M	49	*Cryptococcus* *neoformans*	4	N	*C. neoformans*	N
C20	M	72	Bacterial meningitis	25	N	N	N
C21	M	79	Bacterial meningitis	23	N	N	N
C22	M	60	Bacterial meningitis	46	N	N	N
C23	M	60	Bacterial meningitis	7	*N. gonorrhoeae*	N	N
C24	M	60	Bacterial meningitis	15	N	N	N
C25	M	65	Meningitis	57	***S. suis***	***S. suis***	***S. suis***
C26	M	38	Bacterial meningitis	37	***S. suis***	***S. suis***	***S. suis***
C27^a^	M	63	Meningitis after surgical tooth extraction	7	*K. pneumoniae*	N	N
C28	F	30	Tuberculomeningitis	13	N	N	N
C29	M	41	Bacterial meningitis	22	N	N	N
C30	M	55	Bacterial meningitis	30	***Aeromonas jandaei***	***Aeromonas sobria***	N

BoldItalic indicates concordance, defined as identical results to the species/genus level, between Nanopore sequencing, CSF culture and blood culture

*CSF* cerebrospinal fluid, *M* male, *F* female, *N* negative

^a^Patients deceased

^b^Fungal infection

## Discussion

This study demonstrated that 16S multiplex sequencing using Nanopore sequencing was able to detect pathogens rapidly and inexpensively at the species level directly from CSF samples in a LMIC setting. Nanopore 16S sequencing was able to identify pathogens that were not detected by routine diagnostic methods. Predominant bacteria in CSF samples were *S. pneumoniae* and *S. suis*. This is consistent with results of a recent study of CSF samples in Vietnam [19]. Furthermore, in Vietnam, *S. suis* has been reported to be the most commonly diagnosed bacterium in bacterial meningitis of adults [34, 35], consistent with our results. *S. suis* is widely distributed in East and Southeast Asia, where there is a high density of pigs. Distinct *S. suis* serotypes are known to be highly virulent and to cause severe infections in pigs and humans [36, 37].

Our results are concordant with previous studies, which showed superior performance of NGS-based detection method in detecting pathogens in patients with central nervous system infections [9, 38, 39]. Implementation of molecular-based method as a complimentary method to conventional culture may improve the sensitivity of pathogen detection as indicated by our study. Cerebrospinal fluid (CSF) and blood cultures have been the diagnostic gold standard; however, cultures are time-consuming. Our findings suggest that ≤ 200 reads per sample generated are sufficient to identify bacteria at the species level within an hour using the Nanopore EPI2ME cloud. Rapid identification of bacterial pathogens in bloodstream infections within 6–12 h can accelerate the initiation of appropriate treatment to improve the clinical outcome [40–42].

Oxford Nanopore Technologies (ONT) NGS system is gaining attention as it has several advantages compared to other sequencing platforms. It is a portable device, requires manageable financial investment and low maintenance costs. Moreover, it provides a rapid turnaround time with user-friendly bioinformatics pipelines [12]. Multiplexing sequencing using the MinION system will considerably reduce the costs per sample. For example, the cost of sequencing to detect the bacterial pathogen in any given sample using Oxford Nanopore's 16S barcoding kit is $10. In addition, there is no need for annual instrument maintenance or the use of a bioinformatician, as the data can be analysed directly on Oxford Nanopore's EPI2ME cloud data analysis platform. This additionally reduces the overall cost of maintaining and sustaining Nanopore NGS in LMICs. Furthermore, coinfections or mixed bacterial infections are common in clinics and hospitals and particularly common in the lower respiratory tract and intestinal tract [43, 44]. These coinfections or mixed bacterial infections can be detected using Nanopore NGS shotgun metagenomics or 16S rRNA metagenomic sequencing [45]. As Nanopore NGS has potential to give ultralong reads and real-time data, turnover time can be reduced significantly and thus shorter time-to-result. Additionally, metagenomics approach may provide additional genomic information such as clonal assignment and in-silico analysis of antimicrobial resistance determinants [44].

There are several limitations of the study. First, the study was performed using a small sample size of 30 samples. The small sample size was largely indebted to the limited number of patients with the clinical diagnosis of meningitis admitted to the hospital. Nonetheless, the laboratory parameters of these patients were consistent with the expected laboratory parameters of patients with bacterial meningitis. Furthermore, most studies on the performance of NGS-based detection method were not performed in resource-limited settings. Our study indicates that 16S Nanopore sequencing can be used in resource-limited setting to complement or improve the sensitivity of microbiological diagnostics for bacterial meningitis. In this study, we did not perform metagenomics sequencing. However, as a perspective, metagenomics using nanopore sequencing could be implemented in routine diagnostics for a non-biased pathogen identification and in-silico antibiotic resistance analysis and should be validated in further studies [46].

## Conclusion

The results of this study demonstrate that the 16S multiplex Nanopore sequencing for pathogen detection directly from CSF samples is feasible within six hours without the need of extensive bioinformatics expertise and high-end computing capacities. 16S nanopore sequencing can be a rapid alternative or complementary detection method to conventional microbiological cultures and aid in the timely diagnosis of a causative pathogen in both developed and LMIC countries. Minor optimizations in DNA isolation methodology and increase in sample volumes may be necessary to increase the sensitivity. Acceleration of microbiological diagnostics through the implementation of modern technologies is important and may have a significant impact in improving the clinical management and outcome of severe infections.

## Supplementary Information

**Additional file 1: Table S1.** Nanopore R9.4.1 flow cell sequencing results generated from EPI2ME cloud analysis of complete fastq reads.

## Data Availability

Not applicable. The authors confirm that the data supporting the findings of this study are available within the article and in its supplementary material.

## Abbreviations

16S rRNA: 16S ribosomal RNA
ONT: Oxford Nanopore Technologies
NAT: Nucleic acid test
CSF: Cerebrospinal fluid
LMIC: Low and middle-income countries
NGS: Next-generation sequencing
CT: Computed tomography
MRI: Magnetic resonance imaging
WBC: White blood cells
CNS: Central nerval system
bp: Base pair
