# Supplementary material for: Diagnosis of pathogens causing bacterial meningitis using Nanopore sequencing in a resource-limited setting
Source: Ann Clin Microbiol Antimicrob. 2022 Sep 5;21:39. doi: 10.1186/s12941-022-00530-6 (PMC9443622; doi:10.1186/s12941-022-00530-6)
Supplement: Supplementary file 1 — Additional file 1: Table S1. Nanopore R9.4.1 flow cell sequencing results generated from EPI2ME cloud analysis of complete fastq reads. [file 12941_2022_530_MOESM1_ESM.docx]

**Table S1.** Nanopore R9.4.1 flow cell sequencing results generated from EPI2ME cloud analysis of complete fastq reads.

| **Sample ID** | **Barcode** | **FastQ reads /barcode** | **FastQ reads- length & quality filter** | **FastQ reads-**  **majority species** | **FastQ reads-majority species (%)** | **Majority species** |
| --- | --- | --- | --- | --- | --- | --- |
| C3 | BC01 | 7849 | 5811 | 5498 | 95 | *Streptococcus pneumoniae* |
| C4 | BC14 | 5072 | 4931 | 4122 | 84 | *Streptococcus suis* |
| C7 | BC15 | 6028 | 5843 | 5461 | 93 | *Streptococcus pneumoniae* |
| C8 | BC19 | 13548 | 12979 | 12202 | 94 | *Streptococcus pneumoniae* |
| C10 | BC20 | 12091 | 11662 | 11144 | 96 | *Streptococcus suis* |
| C11 | BC04 | 13357 | 10388 | 9906 | 95 | *Streptococcus suis* |
| C13 | BC18 | 5669 | 5508 | 3423 | 62 | *Enterococcus hirae* |
| C17 | BC07 | 18207 | 16223 | 14840 | 91 | *Streptococcus pneumoniae* |
| C23 | BC08 | 257375 | 227839 | 222223 | 98 | *Neisseria gonorrhoeae* |
| ^$^C25 | BC17 | 13557 | 12737 | 12109 | 94 | *Streptococcus suis* |
| C26 | BC02 | 6777 | 6136 | 5851 | 95 | *Streptococcus suis* |
| C27 | BC09 | 17148 | 16685 | 13397 | 80 | *Klebsiella pneumoniae* |
| C30 | BC17 | 7497 | 7311 | 5333 | 73 | *Aeromonas jandaei* |

^$^Sample was run in a separate sequencing run (not part of the single pool); Percentage (%) of the majority species is calculated based on the reads that passed the quality control and the length filter.
